# Standardization of early drain removal following pancreatic resection: proposal of the “Ottawa pancreatic drain algorithm”

**DOI:** 10.1186/s13037-019-0219-z

**Published:** 2019-12-02

**Authors:** Heather Smith, Fady K. Balaa, Guillaume Martel, Jad Abou Khalil, Kimberly A. Bertens

**Affiliations:** 1 Division of General Surgery, The Ottawa Hospital, University of Ottawa, 725 Parkdale Ave, Ottawa, ON K1Y4E9 Canada; 2 Hepatopancreaticobiliary Subunit, Division of General Surgery, The Ottawa Hospital, University of Ottawa, General Campus, 501 Smyth Road, CCW 1667b, Ottawa, ON K1H 8L6 Canada; 3 Clinical Epidemiology Program, Ottawa Hospital Research Institute, 1053 Carling Ave, Ottawa, ON K1Y4E9 Canada

**Keywords:** Pancreatic resection, Post-operative pancreatic fistula, Drainage

## Abstract

**Background:**

Early drain removal after pancreatic resection is encouraged for individuals with low postoperative day 1 drain amylase levels (POD1 DA) to mitigate associated morbidity. Although various protocols for drain management have been published, there is a need to assess the implementation of a standardized protocol.

**Methods:**

The Ottawa pancreatic drain algorithm (OPDA), based on POD1 DA and effluent volume, was developed and implemented at our institution. A retrospective cohort analysis was conducted of all patients undergoing pancreatic resection January 1, 2016-October 30, 2017, excluding November and December 2016 (one month before and after OPDA implementation).

**Results:**

42 patients pre-implementation and 53 patients post-implementation were included in the analysis. The median day of drain removal was significantly reduced after implementation of the OPDA (8 vs. 5 days; *p* = 0.01). Early drain removal appeared safe with no difference in reoperation or readmission rate after protocol implementation (*p* = 0.39; *p* = 0.76). On subgroup analysis, median length of stay was significantly shorter following OPDA implementation for patients who underwent DP and did not develop a postoperative pancreatic fistula (POPF) (6 vs 10 days, *p* = 0.03). Although the incidence of both surgical site infection and POPF were reduced following the intervention, neither reached statistical significance (38.1 to 28.3%, *p* = 0.31; and 38.1 to 28.3%, *p* = 0.31 respectively).

**Conclusions:**

Implementing the OPDA was associated with earlier drain removal and decreased length of stay in patients undergoing distal pancreatectomy who did not develop POPF, without increased morbidity. Standardizing drain removal may help facilitate early drain removal after pancreatic resection at other institutions.

## Background

Pancreatic resection is the mainstay treatment for resectable pancreatic malignancies, as well as certain benign and premalignant pancreatic disorders [1]. Surgical resection of the pancreas most commonly includes either pancreaticoduodenectomy (PD) or distal pancreatectomy (DP). Improvements in perioperative management have reduced surgical mortality for these patients. Nevertheless perioperative morbidity remains high, in the range of 20–50% at high volume centers [2–5]. Postoperative pancreatic fistula (POPF) is the most frequently cited factor contributing to morbidity. This occurs due to a disruption of the pancreatic anastomosis following PD or a leak from the transected pancreas in DP. The resulting leakage of pancreatic effluent can lead to significant morbidity characterized by deep organ space infection, hemorrhage, end organ failure, and even death [6–9]. The International Study Group on Pancreatic Fistula defined POPF as an amylase level in the drain fluid three times higher than the upper normal serum value on or after post-operative day 3 (POD3) [10]. The clinical impact of a POPF was initially classified by the International Study Group on Pancreatic Fistula as Grade A, B and C, however they have more recently reclassified grade A fistulas as biochemical leaks, which are no longer considered a POPF [7, 10]. Similarly, in this study POPF is defined as Grade B and C pancreatic fistulas, also know as clinically relevant POPF.

At many institutions, including the study center, intraoperative drains are routinely placed in the pancreatic resection bed [11]. Intraoperative placement of drains together with the timing of their removal after both PD and DP have been extensively studied and debated in the surgical literature [11–18]. Surgical drain placement allows for early detection of POPF and drainage of pancreatic effluent, should POPF develop, thereby mitigating the clinical severity and morbidity [17, 19, 20]. Nevertheless, the majority of patients do not develop a POPF, and drains may contribute to ascending infections or anastomotic erosion which has propelled interest in drain omission or early removal [11, 13, 14].

In the case of PD, drain omission has not been well supported in literature, and was correlated to increased mortality in a large multicenter trial [17, 21]. However, selective drain omission and early drain removal in those deemed low risk of a POPF after PD has been shown to be safe and beneficial [11, 15]. Drain omission after DP has been shown to have similar outcomes to routine drain placement in a multi-center trial [22]. Considering the DP transection margin is sterile, selective drain omission may prove beneficial, however currently, drain placement remains recommended [21].

Early selective drain removal after both PD and DP is advantageous and various strategies have been proposed to facilitate this. These included the fistula risk score after PD and drain amylase level after both PD and DP. The fistula risk score is a four-factor score that takes into account gland texture, pathology, pancreatic duct diameter, and intraoperative blood loss to categorize patients from negligible risk to high risk for POPF development. The fistula risk score has been validated in multiple populations of patients undergoing PD [19, 23, 24]. Low post-operative day 1 drain fluid amylase (POD1 DFA) has been discussed by many studies as a predictor for POPF and therefore an indicator for safe drain removal [11, 12, 15, 16, 19, 25–28]. Throughout the literature, the threshold value of POD1 DFA that is used to guide early drain removal is variable, with values ranging from 100 to 5000 U/L [12, 15, 29–34]. A recent review and pooled meta-analysis demonstrated a greater sensitivity for predicting POPF with a drain amylase level cutoff of <1000u/L when compared to higher cutoffs of <5000u/L (87% vs 82%). Similarly, Bertens et al. found that a more conservative cutoff level of 600u/L demonstrated greater sensitivity than a cutoff of <5000u/L (94% vs 33%) [19]. Sensitivity is particularly important to avoid mislabeling patients with a POPF as having no pancreatic leak because, these patients are exposed to the risks associated with drain placement at the time of surgery, but do not gain the benefit of early diagnosis and management of their POPF [9, 13]. Given the established benefit of early drain removal, and improved sensitivity of a lower amylase level cutoff, we sought to implement and assess a standardized protocol to facilitate early drain removal at our institution guided by POD1 DFA <600u/L and low output volume. We did this by implementing a standardized algorithm for the management of operatively placed drains post pancreatectomy across an academic hepatopancreaticobiliary surgical practice, and to track outcomes with regards to this intervention.

## Methods

**Fig. 1:**
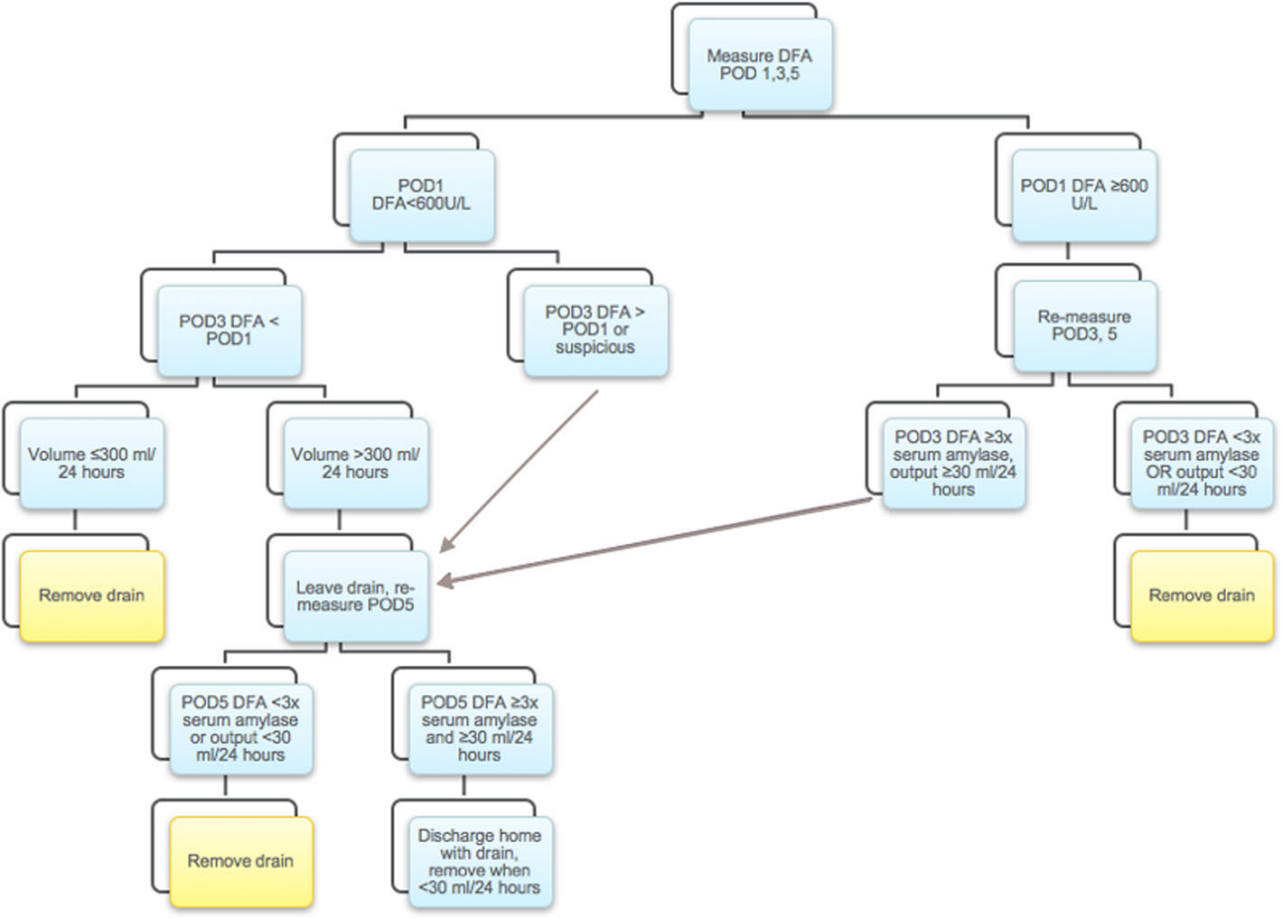
Ottawa pancreatic drain algorithm

After a comprehensive literature review pertaining to risk factors for POPF and cutoff levels of drain fluid amylase, the Ottawa pancreatic drain algorithm (OPDA) was developed at our institution as outlined in Fig. 1. In accordance with the protocol, drains would be removed as early as POD3 based on daily drain output volume and drain fluid amylase levels on POD1 and POD3. Prior to implementation of the protocol, operatively placed drains were managed at an individual surgeon’s discretion. Individual surgeons had a divergent practice; using differing threshold values of POD5 drain fluid amylase and effluent volume to guide removal.

After discussing the indication and feasibility with the Surgical Quality Improvement team, the protocol was approved and implemented in December of 2016. To assess usability, feedback was obtained from the surgical care team and quality improvement team during the 3 months following implementation.

To assess the impact of protocol implementation, a retrospective cohort analysis was completed comparing surgical outcomes before and after implementation of the protocol in all patients undergoing either PD or DP for any indication. With institutional approval by the Research Ethics Board (Protocol # 20170954-01H), data were collected retrospectively for all eligible patients between January 1, 2016 and October 30, 2017. A washout period of one month prior and post implementation of the protocol (November and December 2016) was exercised to minimize the impact of the learning curve. Data were acquired from the American College of Surgeons (ACS) National Surgical Quality Improvement Program (NSQIP). Our center belongs to the hepatopancreaticobiliary ACS-NSQIP Collaborative, collecting pancreatectomy specific Procedure Targeted data [35]. The primary outcome was the median postoperative day on which the last operatively placed drain was removed. A power calculation was conducted for the primary outcome of mean duration of drain placement prior to initiation of the study. A total of 88 patients were needed to provide 80% power to detect a 3-day decrease in the primary outcome with a significance level of 0.05. Secondary endpoints included the proportion of patients discharged from hospital with surgical drains in place, the proportion of patients with a drain on POD30, the proportion of patients requiring percutaneous drain placement, the incidence POPF, the incidence of surgical site infection (SSI), and specifically incidence of organ space SSI. ACS-NSQIP classifies POPF according to their own definition, which does not align completely with the International Study Group on Pancreatic Fistula [35]. Using the ACS-NSQIP variable for POPF, some Grade A fistulas were included. A manual chart review was conducted of all patients with regards to POPF, and the data was coded in accordance with the International Study Group on Pancreatic Fistula definition of Grade B and C POPF [7]. All other outcomes were defined according to the ACS-NSQIP coding manual. Accuracy of the data were verified by manual chart review conducted by authors HS and KB. Non-parametric outcomes were assessed using Mann-Whitney U test, while parametric outcomes were assessed by chi-squared test and Student’s T test using Stata 15.1(©StataCorp, College Station, TX). Multivariate analysis was undertaken using logistic regression for binary outcomes, and linear regression for continuous outcomes. With regards to the primary outcome of drain duration, multivariate analysis was adjusted for time period (pre and post OPDA implementation), type of surgery, organ space SSI and POPF.

## Results

The OPDA was implemented in all patients undergoing pancreatic resection (both PD and DP) between December 2016 and October 30, 2017 at The Ottawa Hospital. Structured feedback with nursing, attending surgeons and surgical residents did not identify any barriers in implementation or usability, and confirmation that the protocol was being used in all eligible patients.

A total of 95 patients were included in this retrospective cohort analysis: 42 prior to implementation of the OPDA and 53 after implementation. This included 65 patients who underwent PD (35 pre-implementation, and 31 post-implementation) and 29 who underwent DP (7 pre-implementation and 22 post implementation). Data pertaining to patient demographics, comorbidities and perioperative factors were compared between the pre-implementation and post-implementation groups, as outlined in Table 1. The pre-intervention group had fewer smokers (3 (7.1%) vs 14 (26.4%), *p* < 0.001), a greater proportion with an ASA score > 2 (40 (95.2%) vs 35 (66%), *p* < 0.001) and fewer patients who underwent distal pancreatectomy (16.6% vs 41%, p < 0.001) when compared with the post-intervention group. The overall incidence of POPF was 32.6% (34.8% for PD, and 27.5% for DP) and was unchanged after the OPDA was implemented (overall 16 (38.1%) pre-intervention vs 15 (28.3%) post-intervention, *p* = 0.312).

**Table 1:** Comparison of demographic and surgical data of study cohort

Demographics	Pre-intervention	Post-intervention	*p* value
	*n* = 42	*n* = 53	
Male gender, N (%)	15 (35.7)	23 (43.3)	0.101
BMI, mean (SD)	26.1 (5.1)	28.1 (6.1)	0.088
Age, mean year (SD)	67 (12.3)	63 (13.1)	0.101
Comorbidities, n (%)			
Diabetes	6 (14.2)	9 (16.9)	
Smoker	3 (7.1)	14 (26.4)	< 0.01
Dyspnea	1 (2.3)	0	0.312
Chronic Obstructive Pulmonary Disease	0	4 (7.5)	
Congestive Heart Failure	0	1 (1.9)	
Hypertension	18 (42.8)	25 (47.7)	0.312
Renal Failure	0	0	
Corticosteroid Use	1 (2.3)	1 (1.9)	1
ASA score > 2	40 (95.2)	35 (66)	< 0.01
Surgery, n (%)			
Pancreaticoduodenectomy (PD)	35 (83.3)	31 (58.4)	< 0.01
Distal Pancreatectomy	7 (16.6)	22 (41)	< 0.01
Laparoscopic DP	2 (28.6)	7 (13.2)	0.883
Pathology			
Adenocarcinoma or Pancreatitis, n (%)	19 (45.2)	28 (52.8)	0.015
Other, n (%)	23 (53.5)	25 (47.1)	0.015
duct diameter, median, mm (range)	4 (1–12)	4 (1–10)	
blood loss, median, mL (range)	450 (50–1600)	350 (50–1500)	
Fistula Risk Score, median	3 (2.75)	3 (4)	

Standard Deviation (SD)

**Fig. 2:**
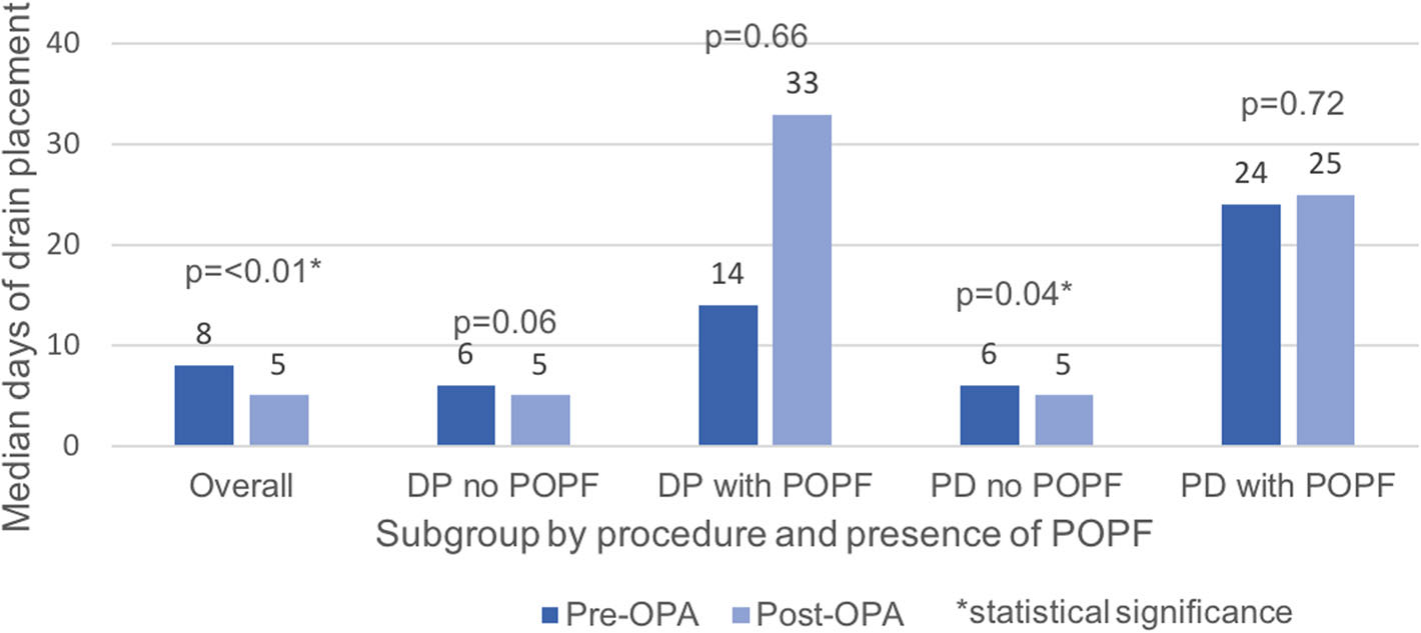
Duration of operatively-placed drains, by surgery type and presence of POPF

The primary outcome was the number of days that operatively placed drains remained in situ (Table 2). Following implementation of the OPDA, drains were removed a median of 3 days earlier (8 vs. 5 postoperative days, *p* = 0.01 in the pre-OPDA and post-OPDA groups respectively) (Fig. 2). On subgroup analysis by surgery type and development of POPF, there was a significant decrease in the median number of days drains were in situ in those undergoing PD not complicated by POPF (6 vs 5 days, *p* = 0.04). Patients that had a DP and did not develop POPF had a median decrease in drain duration of 2 days, however this failed to reach statistical significance (6 vs. 4, *p* = 0.06) (Table 3). In those patients who developed POPF, implementation of the OPDA did not affect when drains were removed.

**Table 2:** Comparison of primary outcome, duration of operatively-placed drains, by surgery type and presence of POPF

	Pre-intervention (median, days)	Post-intervention (median, days)	*p* value
Distal pancreatectomy (DP)			
without POPF	6	4	0.06
with POPF	14	33	0.66
Pancreaticoduodenectomy (PD)			
without POPF	6	5	0.04
with POPF	24	25.5	0.73

**Table 3:** Univariate analysis of outcomes

	Pre-intervention	Post-intervention	*p* value
Presence of Drain			
Drain at discharge (%)	26.2	20.8	0.53
Drain at POD30 (%)	12.2	13.5	0.86
Perioperative Morbidity			
POPF (%)	38.1	28.3	0.31
Pancreaticoduodenectomy	37.1	32.3	0.68
Distal Pancreatectomy	42.9	22.7	0.30
Overall SSI (%)	38.1	28.3	0.31
Pancreaticoduodenectomy	45.7	45.2	0.96
Distal Pancreatectomy	0	4.5	0.57
Organ space SSI (%)	31.0	20.8	0.26
Pancreaticoduodenectomy	37.1	32.3	0.68
Distal Pancreatectomy	0	4.5	0.57
Reoperation (%)	2.3	7.5	0.26
Readmission (%)	14.6	17.0	0.76
Percutaneous drain insertion (%)			
Pancreaticoduodenectomy	11.4	16.1	0.58
Distal Pancreatectomy	14.3	4.5	0.38
LOS (median days) (%)			
Pancreaticoduodenectomy	10	12	0.28
Distal Pancreatectomy	8	6	0.17

Univariate analysis did not reveal a significant improvement in perioperative morbidity following implementation of the OPDA with regards to POPF (*p* = 0.31), overall SSI (p = 0.31), or organ space SSI (*p* = 0.26) (Table 3). The frequency of POPF in patients undergoing DP decreased from 42.9 to 22.7% following implementation of the OPDA, however, this failed to reach significance (*p* = 0.30). In those patients who did not develop a POPF, the rate of organ space SSI was lower following implementation of the OPDA, but this did not reach statistical significance (19.2% vs. 7.9%, *p* = 0.18). Furthermore, there was no significant difference in the proportion of patients with a drain at discharge (26.2% vs. 20.8%, *p* = 0.53), or on POD 30 (12.2% vs. 13.5%, *p* = 0.86), this remained true even when the analysis was restricted to those without a POPF (7.7% vs. 7.9%, *p* = 0.98), (data not shown). Similarly, likelihood of re-intervention remained unchanged including risk of percutaneous drain insertion (*p* = 0.58), re-operation (*p* = 0.26), and readmission (*p* = 0.76) (Table 3). Length of stay (LOS) was not significantly reduced in the univariate analysis for the overall cohort (Table 3). On subgroup analysis, median LOS was significantly shorter following OPDA implementation for patients who underwent DP and did not develop a POPF (6 vs 10 days, *p* = 0.03). In all other subgroups by surgery type and presence of POPF, LOS remained unchanged.

On multivariate analysis adjusting for implementation of the OPDA, type of surgery, organ space SSI and POPF, only the presence of POPF was significantly associated with drain duration in patients undergoing pancreatectomy (*p* < 0.01) (Table 4). Moreover, using multivariate logistic regression, OPDA implementation had no significant association with the likelihood of drain presence at POD 30 (OR 2.0, *p* = 0.37, confidence interval (CI) 0.45–9.41), or a drain at time of discharge (OR 1.0, *p* = 0.92, CI 0.32–3.61) (data not shown).

**Table 4:** Multivariate linear regression of impact on duration of operatively-placed drains of implementation of the OPDA on patients undergoing pancreatectomy

Variable	Odds Ratio	*p* value	Confidence Interval
OPDA	1.7	0.59	−4.60-8.07
Pancreaticoduodenectomy (ref. distal pancreatectomy)	2.7	0.45	−4.40-9.77
Organ space SSI	0.50	0.90	−7.64-8.64
POPF	23.5	< 0.01	16.43–30.63

Implementation of the OPDA was not associated with a decrease of SSI following pancreatectomy, however, the type of surgery (PD vs. DP) was associated with POPF development (Table 5). The results were the same when organ space SSI was considered in isolation (Table 5). The effect of the implementation of OPDA on the odds of developing POPF was analyzed using a separate multivariate analysis for DP and PD. In the case of PD, the analysis was adjusted by a composite of the fistula risk score, whereas for DP, the analysis was adjusted by the pancreatic gland texture. Implementation of OPDA was not significantly associated with POPF in neither PD (OR 0.8, *p* = 0.67, CI 0.28–2.24) nor DP (OR 0.4, *p* = 0.31, CI 0.05–2.52).

**Table 5:** Multivariate logistic regression for SSI among patients undergoing pancreatectomy

Variable	Odds Ratio	*p* value	Confidence Interval
*Overall SSI*			
OPDA	0.8	0.71	0.23–2.72
Pancreaticoduodenectomy (ref. distal pancreatectomy)	70.0	< 0.01	6.34–772.80
Preoperative Biliary Stent	0.6	0.36	0.18–1.88
Preoperative weight loss	0.1	0.12	< 0.01–1.80
Diabetes	0.6	0.49	0.16–2.39
Smoking	2.7	0.26	0.48–15.64
POPF	8.7	< 0.01	2.51–29.87
*Organ Space SSI*			
OPDA	0.5	0.36	0.14–2.06
Pancreaticoduodenectomy (ref. distal pancreatectomy)	36.7	< 0.01	3.47–388.84
Preoperative Biliary Stent	0.5	0.27	0.14–1.73
Preoperative weight loss	0.2	0.25	0.01–3.56
Diabetes	1.2	0.82	0.30–4.53
Smoking	3.2	0.20	0.53–19.50
POPF	9.7	< 0.01	2.90–32.74

Complications associated with early drain removal were also examined. Using a multivariate analysis and controlling for surgery type, fistula risk score, age and ASA score, there was no difference in the odds of percutaneous drain insertion (OR 1.4, *p* = 0.61, CI 0.39–4.89), readmission (OR1.1, *P* = 0.89, CI 0.33–3.58) or reoperation (OR 3.9, *p* = 0.24, CI 0.39–39.62) following implementation of the OPDA.

## Discussion

POPF is the greatest contribution to morbidity following pancreatic resection. Intraperitoneal drains are routinely placed at the time of pancreatectomy to facilitate early diagnosis and management of a POPF by facilitating drainage of pancreatic effluent. However, there is increasing evidence to suggest that when a patient is deemed to be low risk of POPF, drains should be removed early in the post-operative period [11, 12, 15, 16, 19, 25–28]. Drain fluid amylase on POD 1 has been cited as a sensitive predictor for the development of POPF, and an appropriate indicator for when early drain removal is appropriate [12, 13]. A broad range of threshold values have been employed to predict POPF development. A conservative threshold of < 600 u/L has been determined to have appropriate sensitivity to predict POPF, thus avoiding inappropriate early drain removal in patients with a clinically relevant POPF [19, 32]. This study verified that a standardized pancreatic drain algorithm could be successfully implemented across an academic, tertiary care hospital without significant barriers to uptake. Furthermore, the OPDA, facilitated safe, accelerated drain removal by a median of 3 days, when compared to previous routine practice. Accelerated drain removal is, in itself, a formidable accomplishment. Intra-abdominal drains necessitate additional nursing care on surgical wards, complicate discharge planning, and increase the likelihood of requiring community nursing care for assistance in drain management following discharge. This study also demonstrated a shorter LOS in patients undergoing DP who did not develop POPF following implementation of the OPDA (*p* = 0.03). The OPDA was intended to facilitate early removal of operatively placed drains in those who do not develop a POPF following pancreatectomy. A pancreatic leak necessitates that drains remain in place until the effluent dries up, and therefore introduction of a drain algorithm would not be expected to impact the time to removal in patients with POPF.

Concerns have been raised regarding amylase-guided drain removal due to the risk of missing a latent POPF. Latent POPFs account for 18% of all POPFs and are defined by initial drain amylase and output volume within normal range, followed by subsequent development of a POPF [10, 20]. Latent fistulas have been found to be more severe, and require more aggressive intervention than early fistulas [20]. Consequently, prompt drain removal, exclusively directed by amylase level and volume, may contribute to delayed diagnosis and uncontrolled latent fistula [20]. Nevertheless, in this study, there was no increase in percutaneous drainage, hospital readmission or reoperation to suggest a preponderance of missed latent fistulas with early drain removal.

The current study suggests that implementation of a protocol to guide early drain removal after pancreatic resection is both feasible and safe, but does not necessarily improve outcomes. We observed no significant difference in the frequency of POPF, overall SSI, or organ space SSI. Expedient removal of surgically placed drains has been postulated to reduce the risk of ascending infection. In addition, omission of operatively placed drains has been shown to have no negative impact on risk of POPF or overall surgical morbidity and mortality [17]. This study was powered to a primary outcome of time to drain removal, and therefore may have been underpowered to detect a difference in postoperative morbidity. For instance, a study of 4992 patients would be required to power a study demonstrating a 10% relative reduction in SSI with beta of 1–0.8, alpha of 0.05. Although this study fails to demonstrate a significant improvement in outcomes following early drain removal in patients undergoing DP, the risk of POPF trended downwards (42.8 to 22.7%) following introduction of the OPDA. The inability to reach statistical significance may be due to the relatively small proportion of patients undergoing DP in our cohort. Furthermore, other prospective studies have demonstrated a significant reduction in POPF and related complications with earlier drain removal. In a prospective trial of 114 patients by Bassi et al. found that early drain removal in low risk patients was associated with a significant reduction in POPF (*p* = 0.0001) as well as other abdominal complications (*p* = 0.002), pulmonary complications (*p* = 0.007), LOS (*p* = 0.018) and hospital cost (*p* = 0.02) [12]. Similarly, Kawai et al. demonstrated a decrease in morbidity associated with early drain removal after pancreatic resection (*p* = 0.004) [31]. Although this study fell short of statistically demonstrating significant improvements in POPF or SSI, there was a greater than 50% reduction in organ space SSI before and after implementation of the OPDA (both PD and DP) and significant decrease in LOS after DP without POPF (*p* = 0.03). Furthermore, we established that the OPDA could be swiftly and successfully implemented, and safely utilized to guide early drain removal.

The generalizability of the results is limited by the retrospective nature of the study and involvement of a single institution. Furthermore, the OPDA did not account for fistula risk score, patient age, leukocytosis, or c-reactive protein level despite these having been shown to be independent predictors of POPF development in other studies [20, 30, 36, 37]. It was not within the scope of this study to contribute to the diagnostic criteria of POPF. Rather, the aim was to provide an algorithm that can be easily employed to guide operative drain management post pancreatectomy. Further research is required to address whether the addition of other risk factors would improve the accuracy of the OPDA. As such, we advocate for surgeons to consider standardizing drain management at their institution, and that the protocol itself maybe unique to each institution; the OPDA is one such protocol that can be utilized.

## Conclusion

In conclusion, this study demonstrates the successful implementation of the OPDA to standardize drain removal after pancreatic resection using POD1 DFA cutoff of <600u/L and drain effluent volume less than 300 ml. This resulted in earlier drain removal by a median of 3 days, with no increase in latent POPF. The authors recommend institutional standardization of post pancreatectomy drain management to facilitate early drain removal as demonstrated in this study using an algorithm guided by DFA. Further research is required to understand if the protocol may significantly improve outcomes and if there is a role for integrating other risk factors known to contribute to POPF.

## Data Availability

The data generated and analyzed during this study are not publicly available due to patient confidentiality but are available from the corresponding author on reasonable request.
